# Global Diversity of Marine Isopods (Except Asellota and Crustacean Symbionts)

**DOI:** 10.1371/journal.pone.0043529

**Published:** 2012-08-31

**Authors:** Gary C. B. Poore, Niel L. Bruce

**Affiliations:** 1 Museum Victoria, Melbourne, Victoria, Australia; 2 Museum of Tropical Queensland and School of Marine and Tropical Biology, James Cook University, Townsville, Queensland, Australia; 3 Department of Zoology, University of Johannesburg, Auckland Park, South Africa; Technical University of Denmark, Denmark

## Abstract

The crustacean order Isopoda (excluding Asellota, crustacean symbionts and freshwater taxa) comprise 3154 described marine species in 379 genera in 37 families according to the WoRMS catalogue. The history of taxonomic discovery over the last two centuries is reviewed. Although a well defined order with the Peracarida, their relationship to other orders is not yet resolved but systematics of the major subordinal taxa is relatively well understood. Isopods range in size from less than 1 mm to *Bathynomus giganteus* at 365 mm long. They inhabit all marine habitats down to 7280 m depth but with few doubtful exceptions species have restricted biogeographic and bathymetric ranges. Four feeding categories are recognised as much on the basis of anecdotal evidence as hard data: detritus feeders and browsers, carnivores, parasites, and filter feeders. Notable among these are the Cymothooidea that range from predators and scavengers to external blood-sucking micropredators and parasites. Isopods brood 10–1600 eggs depending on individual species. Strong sexual dimorphism is characteristic of several families, notably in Gnathiidae where sessile males live with a harem of females while juvenile praniza stages are ectoparasites of fish. Protandry is known in Cymothoidae and protogyny in Anthuroidea. Some Paranthuridae are neotenous. About half of all coastal, shelf and upper bathyal species have been recorded in the MEOW temperate realms, 40% in tropical regions and the remainder in polar seas. The greatest concentration of temperate species is in Australasia; more have been recorded from temperate North Pacific than the North Atlantic. Of tropical regions, the Central Indo-Pacific is home to more species any other region. Isopods are decidedly asymmetrical latitudinally with 1.35 times as many species in temperate Southern Hemisphere than the temperate North Atlantic and northern Pacific, and almost four times as many Antarctic as Arctic species. More species are known from the bathyal and abyssal Antarctic than Arctic GOODS provinces, and more from the larger Pacific than Atlantic oceans. Two areas with many species known are the New Zealand-Kermadec and the Northern North Pacific provinces. Deep hard substrates such as found on seamounts and the slopes are underrepresented in samples. This, the documented numbers of undescribed species in recent collections and probable cryptic species suggest a large as yet undocumented fauna, potentially an order of magnitude greater than presently known.

## Introduction

Isopod crustaceans occupy all habitats, from the desert to the deep sea with the exception of terrestrial Antarctica. Marine species (those that breed in marine or estuarine habitats) are known from the supralittoral and intertidal to depths in excess of six kilometres. Isopods are a highly diverse group of crustaceans, with more than 10,300 species known to date, approximately 6,250 of these being marine or estuarine. In the groups under discussion here (about half the species) the vast majority of species are known from depths of less than 1000 metres.

The Isopoda is one of the orders of peracarid crustaceans, that is, those that brood their young in a marsupium under the body. They are uniquely defined within Peracarida by the combination of one pair of uropods attached to the pleotelson and pereopods of only one branch. Marine isopods are arguably the most morphologically diverse order of all the Crustacea. Many species have a dorsoventrally compressed body shape, usually with a vaulted dorsum, notably the Cymothoida and the family Sphaeromatidae. The Anthuroidea exhibit bodies that are extremely elongate and cylindrical (vermiform) while the Serolidae and some Sphaeromatidae are strongly flattened (scale-like). The Valvifera and Sphaeromatidae may display a high degree of ornamentation in the form of spines and nodules. Most are bilaterally symmetrical but some parasitic cymothoids are variously twisted. Sexual dimorphism in body shape and mouthparts is common in many families.

## Methods

**Figure 1:**
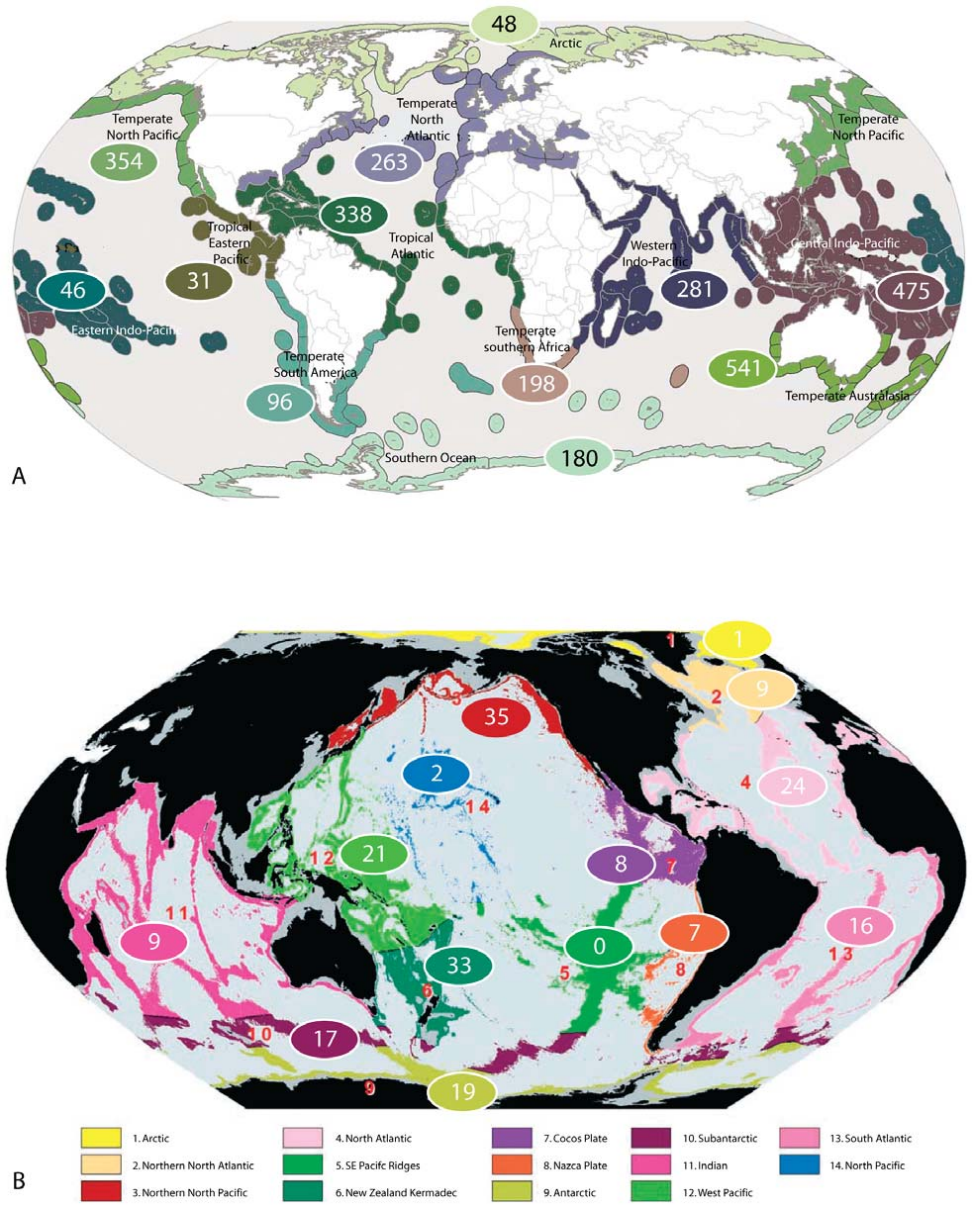
Representative marine isopod forms. Cirolanidae: a, *Bathynomus* sp. b, *Natotolana woodjonesi*. c, *Cirolana* sp. Aegidae: d, *Creniola laticauda* on sea dragon. Gnathiidae: e, f, *Elaphognathia ferox* (male and female). Anthuridae: g, *Mesanthura astelia*. Paranthuridae: h, *Paranthura* sp. Limnoriiidae: i, *Limnoria* sp. j, *Lynseia himantopoda*. Chaetiliidae: k, *Austrochaetila capeli*. Holognathidae: l, *Cleantis phryganaea*. Idoteidae: m, *Batedotea collingei*. Antarcturidae: *Antarcturus* sp. Arcturidae: o, *Neastacilla tharnardi*. Serolidae: p, *Serolina delaria*. Plakarthriidae: q, *Plakarthrium australiensis*. Sphaeromatidae: r, *Maricoccus brucei*. s, *Zuzara venosa*. t, *Cerceis tridentata*.

**Figure 2:**
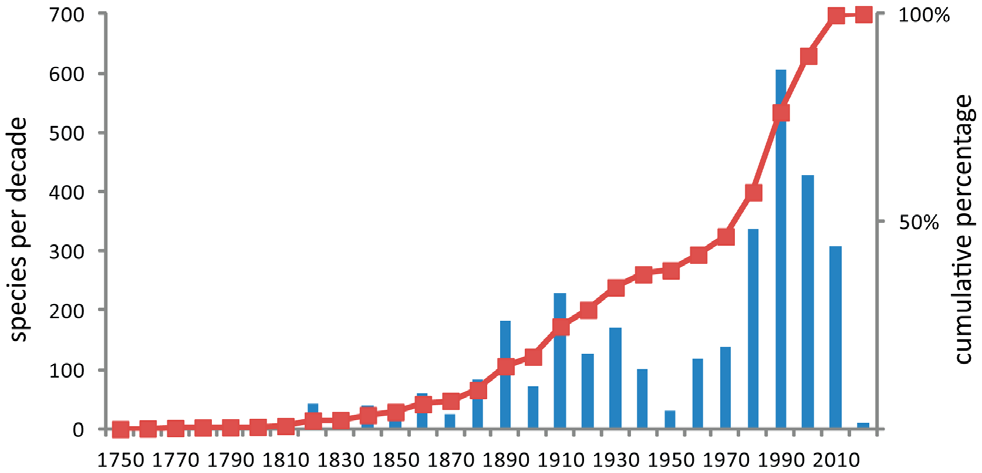
Numbers of marine Isopoda (except Asellota and crustacean symbionts) in biogeographic regions. A. In 12 MEOW biogeographic realms for 2851 species with minimum depths of <800 m. The few species known to occur in >1 realm are assigned only once on the basis of type locality. B. In 14 GOODS lower bathyal provinces for 202 species with minimum depths >800 m. More detailed data for families are given in Tables 2 and 3. No species are known to occur in >1 province.

This contribution reviews the diversity of the marine Isopoda exclusive of the Asellota (planned by G.D.F. Wilson for this journal) and those isopods that are symbionts of marine crustaceans, namely the Bopyroidea and Cryptoniscoidea [1]. The taxa with numbers of species are listed in Table 1 and representative taxa are shown in Figure 1. Therefore all text relates to cymothooidean superfamilies Cymothooidea and Anthuroidea, and suborders Limnoriidea, Valvifera and Sphaeromatidea. Historic references are not cited but can be readily accessed through references cited here and World List of Marine, Freshwater and Terrestrial Isopod Crustaceans available in two formats, one hosted at the Smithsonian Institution [2] and the other as part of WoRMS (World Register of Marine Species, hosted at the Belgian Institute of Marine Science (VLIZ)) [3]. Data sources are current at the end of 2010. The *Sphaeromatid Isopods Worldwide* resource [4] was also consulted. The primary data source is the WoRMS database, augmented for general biology and ecology by our own experience with the fauna and literature. To appreciate how marine isopods are distributed globally, species records have been allocated to one or other of two biogeographic schemes according to bathymetric records: (1) realms of the Marine Ecoregions of the World (MEOW) which is a bioregionalization of coastal and shelf areas [5] (Table 2, Fig. 2A); or (2) lower bathyal provinces (800–3000 m) of the Global Open Oceans and Deep Seabed (GOODS) biogeographic classification [6] (Table 3, Fig. 2B). All records from the intertidal down to 800 m depth have been allocated to the MEOW scheme as this probably better reflects their patterns than the lower bathyal provinces of the GOODS scheme; the few records from depths >3000 m use the GOODS categories. With very few exceptions known distributions fall within only one realm or province. The names of the 12 MEOW realms and 14 GOODS provinces can be found in the tables. Analysis of the differences in numbers of species between regions (MEOW realms or GOODS provinces) must be tempered with an appreciation of historical differences in sampling effort (see next section). Only gross generalisations can be made on the basis of these data.

**Table 1:** Families of Isopoda with marine representatives: numbers of marine families, genera and species.

SuborderSuperfamily Family	Numbers of taxa		
	families	genera	Species
**Phoratopodidea**	**1**	**1**	**1**
Phoratopodidae	1	1	1
**Cymothoida**	**15**	**175**	**1723**
**Cymothooidea**	**9**	**119**	**1152**
Aegidae*		7	147
Anuropidae		1	10
Barybrotidae		1	1
Cirolanidae*		44	412
Corallanidae		7	74
Cymothoidae*		34	280
Gnathiidae		12	205
Protognathiidae		1	2
Tridentellidae		1	21
**Anthuroidea**	**6**	**56**	**571**
Antheluridae		3	18
Anthuridae*		24	267
Expanathuridae		7	58
Hyssuridae		6	39
Leptanthuridae*		10	96
Paranthuridae		6	93
**Limnoriidea**	**3**	**5**	**62**
Hadromastacidae		1	3
Keuphyliidae		1	1
Limnoriidae		3	58
**Valvifera**	**11**	**85**	**603**
Antarcturidae		17	116
Arcturidae		15	158
Arcturididae		1	2
Austrarcturellidae		5	45
Chaetiliidae*		12	44
Holidoteidae		3	20
Holognathidae		5	25
Idoteidae*		24	185
Pseudidotheidae		1	4
Rectarcturidae		1	3
Xenarcturidae		1	1
**Sphaeromatidea**	**7**	**124**	**765**
Ancinidae		2	14
Bathynataliidae		3	4
Basserolidae		1	2
Plakarthriidae		1	3
Serolidae		22	109
Sphaeromatidae*		94	619
Incertae sedis			2
Tecticipitidae		1	12
**Totals**	**37**	**379**	**3154**

Families marked * have non-marine/freshwater genera and species not counted in this analysis.

**Table 2:** Distribution of 2851 species of marine isopoda (except Asellota and crustacean symbionts) by family and MEOW realms.

SuborderSuperfamily Family	Arctic	Temperate Northern Atlantic	Temperate Northern Pacific	Tropical Atlantic	Western Indo-Pacific	Central Indo-Pacific	Eastern Indo-Pacific	Tropical Eastern Pacific	Temperate South America	Temperate Southern Africa	Temperate Australasia	Southern Ocean
**Phoratopodidea**											**1**	
Phoratopodidae											1	
**Cymothooidea**	**13**	**101**	**122**	**146**	**131**	**261**	**16**	**19**	**18**	**47**	**148**	**24**
Aegidae	6	15	18	10	3	33	1	4	4	4	27	6
Anuropidae	2		1					2	1		1	1
Barybrotidae					1							
Cirolanidae	1	33	18	59	50	99	4	8	6	33	66	9
Corallanidae			14	17	14	24	1		1	1	2	
Cymothoidae		32	39	41	44	79	6	5	4	2	17	1
Gnathiidae	4	21	32	19	19	26	4		2	7	35	7
Protognathiidae												1
Tridentellidae		3	6		4	1	1		1	2	3	
**Anthuroidea**	**2**	**46**	**40**	**90**	**57**	**108**	**21**	**3**	**11**	**42**	**86**	**24**
Antheluridae		3	2	1	3	1	1			1	2	1
Anthuridae	1	23	21	48	30	64	8	2	2	24	34	5
Expanathuridae		4	1	13	6	11	3		1	3	9	6
Hyssuridae		7		7	3	6	2			2	4	3
Leptanthuridae	1	6		9	8	18	2			9	17	2
Paranthuridae		3	16	12	7	8	5	1	8	3	20	7
**Limnoriidea**	**0**	**7**	**7**	**4**	**6**	**10**	**2**	**0**	**1**	**0**	**20**	**2**
Hadromastacidae					1		1				1	
Keuphyliidae						1						
Limnoriidae		7	7	4	5	9	1		1		19	2
**Valvifera**	**31**	**60**	**109**	**31**	**14**	**10**	**2**	**2**	**27**	**43**	**79**	**84**
Antarcturidae	5		7	1					3	5	1	54
Arcturidae	13	24	31	13	7	2			4	16	24	3
Arcturididae												2
Austrarcturellidae				1		3			2		16	12
Chaetiliidae	3	5	3	4		3			11		7	5
Holidoteidae										15		
Holognathidae		1	7	3				1	3	1	6	3
Idoteidae	10	30	61	9	7	2	2	1	1	6	23	5
Pseudidotheidae									1		2	
Rectarcturidae									1			
Xenarcturidae									1			
**Sphaeromatidea**	**2**	**49**	**76**	**67**	**73**	**86**	**5**	**7**	**39**	**66**	**207**	**46**
Ancinidae		2	5	4		1						
Basserolidae											2	
Bathynatalidae						2						
Plakarthriidae											2	1
Serolidae		1	1	7		6			16	1	20	31
Sphaeromatidae		46	62	56	73	77	5	7	23	65	183	14
Tecticipitidae	2		8									
**Totals**	**48**	**263**	**354**	**338**	**281**	**475**	**46**	**31**	**96**	**198**	**541**	**180**

Total numbers are mapped on to the MEOW biogeographic realms in Fig. 2A. While MEOW realms apply strictly to coastal and shelf waters down to 200 m species have been included here where minimum recorded depths are <800 m; species on the upper slope are not included in the GOODS upper bathyal provinces. No data are available for a small number of known species.

**Table 3:** Distribution of 202 species of marine isopoda (except Asellota and crustacean symbionts) by family and GOODS lower bathyal provinces.

SuborderSuperfamily Family	Arctic	Northern North Atlantic	Northern North Pacific	North Atlantic	Southeast Pacific Ridges	New Zealand-Kermadec	Cocos Plate	Nazca Plate	Antarctic	Subantarctic	Indian	West Pacific	South Atlantic	North Pacific
**Cymothooidea**	**1**	**4**	**5**	**8**	**0**	**15**	**5**	**7**	**2**	**4**	**3**	**7**	**2**	**2**
Aegidae		1		1		4	4	3		1		1		1
Anuropidae										1		1		
Cirolanidae		1		1		5		1		2	1	4		1
Cymothoidae			1											
Gnathiidae	1	2	4	6		6	1	2	1		2	1	2	
Protognathiidae									1					
Tridentellidae								1						
**Anthuroidea**	**0**	**4**	**3**	**10**	**0**	**6**	**1**	**0**	**0**	**0**	**1**	**1**	**3**	**0**
Antheluridae			2	1										
Anthuridae				1		1	1						1	
Expanathuridae						1								
Hyssuridae		1		4										
Leptanthuridae		3	1	3		4						1	1	
Paranthuridae				1							1		1	
**Limnoriidea**						**1**								
Limnoriidae			1			1								
**Valvifera**	**0**	**1**	**23**	**4**	**0**	**6**	**3**	**0**	**13**	**13**	**2**	**5**	**2**	**0**
Antarcturidae			9			3	2		9	10		2	1	
Arcturidae		1	7	1								1		
Austrarcturellidae				2		2			2	1		2	1	
Chaetiliidae			1			1								
Holidoteidae							1		2		2			
Idoteidae			6	1										
Rectarcturidae										2				
**Sphaeromatidea**	**0**	**0**	**4**	**2**	**0**	**5**	**0**	**0**	**4**	**0**	**3**	**8**	**9**	**0**
Ancinidae			2											
Bathynataliidae											2			
Serolidae				1		5			4		1	5	9	
Sphaeromatidae				1								3		
Tecticipitidae			2											
**Totals**	**1**	**9**	**35**	**24**	**0**	**33**	**9**	**7**	**19**	**17**	**9**	**21**	**16**	**2**

Total numbers are mapped on to the GOODS provinces in Fig. 2B. Assignment of species to provinces is based on minimum depth records >800 m down to a maximum of 7000 m (GOODS lower bathyal provinces strictly apply to the range 800–3000 m but numbers beyond this depth are few).

## Results and Discussion

### History of discovery

**Figure 3:**
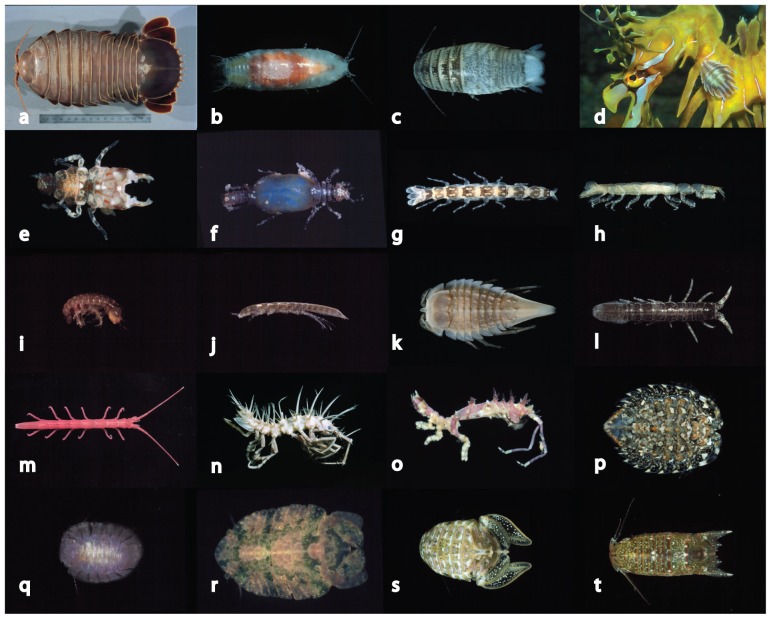
Absolute numbers and cumulative percentage of species of marine Isopoda (3154) published per decade since Linnaeus, 1758.

While the first isopods were named by Linnaeus, the starting point for the history of discovery for marine Isopoda can be thought of as 1840, the date of publication of Milne Edwards' treatise on Crustacea [7]. In the period 1840–1900 progress was erratic, largely reliant on European or North American expertise and the material basis for isopod taxonomy at that time was limited by available collecting methods and also the technical limitations of the equipment used. Outstanding contributions from this era include the global monographs produced by Danish authors J.C. Schioedte and F. Meinert (1879–1884: Cymothoidae, Aegidae and Corallanidae) [8] and the equally outstanding contribution of Hans Jacob Hansen, also Danish, that included his revisions of the Cirolanidae and Sphaeromatidae [9], [10]. British authors, the Reverend T.R.R. Stebbing [11], [12] and E. J. Miers [13], [14], towards the end of the 19th century (carrying on into the 1920s), described many species from the Indo-West Pacific. Beddard, publishing result from the HMS *Challenger* expedition also made a significant contribution [15], [16]. Théodore Monod, in >50 contributions that spanned the Word War II period, made significant marks, one being his monograph of the Gnathiidae [17] and another his review of Cirolanidae [18]. At a regional level the works of Harriet Richardson at the turn of the century made significant and monographic contribution to the isopod fauna of North America [19], while in the early part of the 20th century Keppel Barnard made a huge contribution in documenting South African isopods of which [20], [21] are examples. In Australia the major contributors from this era were T. Whitelegge, W. H. Baker and H.M. Hale [22]. H. F. Nierstrasz, in his contributions to the *Siboga* Expedition, 1923–1941, provided a summary of knowledge to date for the Isopoda [23]. R. J. Menzies (and his collaborators) made a substantial contribution to the isopod fauna of the Americas, principally in the period 1950–1970s; Menzies is perhaps best known for his monograph on the Isopoda of Chile and the Caribbean abyss [24], [25] and his revisions of the Limnoriidae [26] and some valviferan genera [27]. In the modern era, the use of SCUBA and fine-mesh epibenthic sleds (first developed by Theodore Mortensen but later successfully developed for ship use by R.J. Menzies) effectively revolutionised the ability to collect small (1–5 mm long) isopods, particularly from shallow subtidal habitats and the continental shelf and slope respectively. From about the mid-1970s the major contributors (and their students) worked on large collections of this new and rich source of specimens, including (within the taxa under consideration) Angelika Brandt, Niel Bruce, Brian Kensley, Oleg Kussakin, Hans-Georg Müller, Gary Poore and Wolfgang Wägele. Figure 3 shows the clear biphasic discovery of new species, the first strong phase during 1880s–1930s and the second during 1980s–2000, a pattern common for many taxa. The rate of species discovery has slowed since the 1990s. Almost 20% of all known species and almost 10% of genera were described during the 1990s. The rate of description would appear to have slowed but this does not indicate completion of the task (see below).

### Morphology

The isopod body is divided into three regions: a head of fused segments including the first thoracomere; a pereon of 7 segments, usually free; and an abdomen of 5 segments, sometimes fused, plus pleotelson (fused last pleonal segment and telson). The head carries 2 pairs of antennae (antennules and antennae), mandibles, maxillules, maxillae and maxillipeds. The pereon has 7 pairs of pereopods (often but not always similar walking legs; sometimes some are lacking), each of only one branch. The abdomen has 5 pairs of lamellar biramous pleopods sometimes modified, plus a pair of biramous uropods attached to the pleotelson. Females carry eggs, embryos and juveniles in a ventral marsupium derived from the pereopodal coxae (as in all peracarid crustaceans) or in a ventral pouch in some sphaeromatids and cirolanids. Males uniquely bear a pair of stylets on inner edges of the endopods of the second pleopods. Juveniles lack the last pairs of pereopods, hatching as the so-called manca stage, these pereopods appearing and developing in size with successive moults.

Marine isopods range in size from approximately 1 mm (smallest asellotes and anthuroids, bordering on interstitial or meiofauna size) and 2 mm (smallest Gnathiidae, Cirolanidae, Sphaeromatidae) to the largest of all isopods, *Bathynomus giganteus* at over 350 mm [28]). The overwhelming majority of species are in the size range of 3 to 20 mm. Very few isopods exceed 50 mm. Shallow-water species may be cryptically coloured or patterned [29] though such colours are usually lost on preservation. Pigmented patterns are rare but can be persistent and species-specific in genera such as *Mesanthura* (Anthuridae). Chromatophores often contribute to changing patterns and colours within individuals. Isopods such as sphaeromatids and arcturids living on alga or algal turf may be strongly coloured though such colours are generally cryptic, matching for example the red of coralline algae or blue and green of other algae. Some sand-dwelling species such as serolids and *Eurydice* (Cirolanidae) are also cryptically coloured. Fish-parasitic and deep-water species are generally without pattern, or weakly coloured, deep-water species generally pale to red pink (Aegidae, some Cirolanidae) or white to pale tan (others). Isopods are exceptionally diverse in body form (Fig. 1) and variously use body shape, ornamentation and setation as apparent camouflage and defensive strategies. The body of basserolids and some serolids and sphaeromatids is flattened to provide the least profile on sediment or a hard substrate. Several arcturoid valviferan families use their elongate cylindrical body to stand erect from their habitat. Antarcturids in particular are covered in strong spination that could be assumed to be defensive.

The transition from free-living predation to parasitism in the Cymothooidea is described under ‘Feeding’. Associated with this change in feeding mode is an associated change in morphology. Setae become increasingly fewer as the level of parasitism increases, and the body segments become increasingly smooth. The mouth appendages of worm predators (e.g. *Lanocira*, Corallanidae) have piercing and suctorial mouthparts, with the maxillule of *Lanocira* having the form of a large hook, eminently suited to grasping small polychaetes. The feeding habits of some of these carnivorous and scavenging taxa could be seen as a transferable ‘pre-adaption’ to developing a more parasitic feeding method. In Aegidae, Tridentellidae and Cymothoidae the mouthparts form a distinct ‘buccal cone’; typically with the incisory appendages lacking slender setae and the maxilliped, maxilla and maxillule having strongly recurved and hooked robust setae or abrading serrate scales. Pereopod morphology also changes with increasing levels of parasitism – in corallanids the pereopods have relatively few setae but are largely ambulatory. In Aegidae the anterior three pairs of pereopods are prehensile, retain some robust setae, but in some species also display a scraping or ‘spoon-like’ surface. Finally, the obligate parasitic Cymothoidae have strongly recurved dactyli on all pereopods. Free-living cymothoids have well-developed eyes, as do the commensal families, lack of eyes being associated with extreme turbidity or depth. In Aegidae eyes are absent in the mesopelagic genera but can be large; in many species the eyes occupy the entire dorsal surface of the head. In contrast, the parasitic Cymothoidae have large eyes at the paratenic stage but these become smaller in adults, with the gill and buccal-attaching genera having small eyes or eyes covered by thick cuticle that obscures the ommatidia. As would be expected, with increasing level of parasitism motility decreases to the state in adult cymothoids that are unable to leave their final host or to swim or crawl. Gnathiid isopods differ from all others in the Cymothooidea in that it is the juvenile or praniza stage that feeds on fish blood; these pranizas are little modified and retain setose, ambulatory pereopods and setae on their pleopods and usually have large eyes; the mouthparts are incisory and suctorial in structure.

### Relationships and classification

The relationship of Isopoda to other Peracarida has not always been well understood. One thing that is almost universally agreed is that Isopoda are monophyletic. Richer & Scholtz [30] reviewed much of the earliest work in which Isopoda had been various related to Tanaidacea or Amphipoda, or treated as sister taxon to all other peracarid orders. They discussed many morphological traits in detail and concluded on the basis of a cladistic analysis of all malacostracan orders and suborders that Isopoda were more probably sister taxon to Tanaidacea than to any other taxon. Poore [31], also on the basis of a cladistic analysis of morphological characters, concluded that Isopoda were more derived peracaridans than others and sister to Amphipoda, another superabundant group without a carapace or pereopodal exopods. Wilson [32] criticised this result and found some morphological and molecular support for Isopoda and Tanaidacea being similar but not sister-taxa. By his own admission his results were inconclusive but he was supported in part by Tabacaru & Danielopol [33]. Jenner et al. [34] found conflict between morphological and molecular evidence but found little support for the Isopoda-Amphipoda relationship.

Wägele [35] and later Brandt & Poore [36] reviewed earlier hypotheses concerning isopod relationships. Wägele's [35], [37] ‘Hennigian’ treatment and Brusca & Wilson's cladistic analysis [38] both placed Phreatoicidea, Asellota and Oniscidea, all with styliform uropods, at the base of a tree of isopod relationships. They differed in their treatment of these ‘short-tailed’ taxa, Wägele seeing them as polyphyletic and derived from an ancestral ‘flabelliferan’ type while Brusca & Wilson saw this type as derived; they called it the ‘long-tailed group’ whose members possess expanded pereopodal coxal plates and broad uropods. They also differed in their interpretation of relationships within these long-tailed taxa; Wägele dividing them into separate clades, Cymothoida, Sphaeromatidea and Valvifera, while Brusca & Wilson found their constituent families only partially resolved. On the basis of molecular studies, Dreyer & Wägele [39], [40] erected a more inclusive taxon that they called Scutocoxifera by adding the Oniscidea to the former flabelliferan families. Relationships within this clade were reappraised using morphology by Brandt & Poore [36] who largely supported Wägele's classification, if not his evolutionary hypothesis. Wilson's [32] combined morphological and molecular treatment hypothesised several unconventional and conflicting relationships that could not be satisfactorily resolved. Wilson's [32] analysis of the Isopoda using molecular (18S) and morphological data and controversial analytical methods failed to contradict this classification but notably split representatives of the Cymothoida into disparate clades.

Phylogenetic studies at the sub-superfamilial level within the taxa of interest are few. Most examples rely on morphological data and have hypothesised relationships between all isopod taxa [37], between families and genera of Anthuroidea [41], [42], between families of Valvifera [43], phylogeny and biogeography of Corallanidae [44] and Gnathiidae [45], or between genera of Idoteidae (now including Holognathidae and Chaetiliidae) [46] and Aegidae [47] and also within genera of Sphaeromatidae [48] and Cirolanidae [49], [50].

In the decade since Wetzer [51] lamented the absence of molecular studies of isopod relationships several studies have been published. Held and coworkers compared the phylogeny and biogeography of some genera of Serolidae [52], [53] and illuminated cryptic speciation in the Antarctic species *Glyptonotus antarcticus* and *Serolis paradoxa* [54], [55], Wilson suggested that Gnathiidae are not cymothooids [56], Ketmaier et al. have shown that parasitic feeding strategies in Cymothoidae are independently derived [57], Baratti et al. have resolved relationships between freshwater and anchialine stygiobiont species of American and Mediterranean Cirolanidae [58] and Prevornik et al. [59] elucidated the phylogeny and biogeography of stygial freshwater *Monolistra* (Sphaeromatidae).

The suborders with marine taxa now recognised are Phoratopodidea, one family and species, Cymothoida (which includes some parasitic families excluded from our review), Limnoriidea, Valvifera and Sphaeromatidea. Table 1 summarizes the current classification that is a widely agreed compromise derived from recent phylogenetic and taxonomic research.

### Taxonomic diversity

Thirty-seven families are discussed here, ranging in diversity from one to hundreds of genera and species (Table 1). Some have freshwater representatives discussed in a similar context by Wilson [60] and not included here.

The groups considered here contribute around 60% (3154 accepted species in 379 genera in 37 families; Table 1) of all described marine isopods. The suborder Asellota that dominates in the deep sea comprises around 1600 known species, parasitic bopyroids 605 and cryptoniscoids 99 species [1]. Of the five suborders considered here, the Cymothooidea contribute 54% of all species. This suggests the success of the scavenging, parasitic and predatory life-styles [40] but is also partly attributable to the relatively large size of these species (mostly 3–20 mm) as well as their ease of collecting. Some families are monotypic or have few species while others are exceptionally rich in species. The high numbers of species in some families correlates with high morphological diversity and reflects on underdeveloped taxonomies. This has been counteracted recently with the creation of several families where previously there was one or few, notably in Valvifera [43] and Anthuroidea [41]. The Sphaeromatidae with almost 100 genera and 619 known marine species (and ∼65 in fresh water) can be thought of in terms of several genus-groups with distinctive morphologies that could be considered families in future.

Despite more than 160 years of isopod taxonomy and the large number of described species, many more remain to be described. Species yet to be discovered will come from several sources: study of families that have so far failed to attract taxonomic attention; exploration of new regions such as rocky continental slopes; sampling of difficult habitats; and revelations of cryptic species using new (especially molecular) methods.

High species diversity in some families can be attributed to the recent attention of few taxonomists who dedicated time to describing numerous species and systematic studies: Aegidae, Cymothoidae, Cirolanidae (J.C. Schioedte & F. Meinert, H. -J. Hansen, N.L. Bruce), Anthuroidea (J.-W. Wägele, G.C.B. Poore), Sphaeromatidae (D.M Holdich & K. Harrison, N.L. Bruce), and Valvifera (G.C.B. Poore, A. Brandt) while others (H. Richardson, K.H. Barnard, B. Kensley, N. Nunomura, R.J. Menzies) each described 250 or more species without specialising. These families still deserve attention but some others remain poorly understood, e.g., Serolidae, Antarcturidae, Arcturidae. The rate of species discovery in the smallest families and in others appears to have plateaued, e.g., Idoteidae, Holidoteidae, Holognathidae, Chaetiliidae. These families are from shallow easily accessible habitats.

### Fossils

The fossil record is moderately strong for certain suborders of Isopoda such a the Cymothoida (Cirolanidae) and Sphaeromatidea, Feldmann and Rust [61] listing 26 species of *Palaega*, many of which those authors regarded as not belonging to the genus sensu stricto. Unfortunately this uncertainty applies to most fossil isopods [62], which cannot be assigned to a extant families or genera (see several papers by Feldmann), and so do not fit into modern classification. Exceptions are some fine recognizable fossils of *Bathynomus*, and also of unambiguous Sphaeromatidae [63], [64]. Bowman [65] showed that *Palaega lamnae* could be classified equally as a cirolanid or cymothoid, and most fossils are placed to a ‘best fit’ rather than from diagnostic morphological characters. Given these limitations the fossil record at contributes minimally to our understanding of isopod diversity, biogeography and evolution. Feldmann & Charbonnier [66] demonstrated the difficulty of assigning fossils to taxon with the case of a fossil described in a genus of slipper lobster being in fact a species of *Cirolana*.

### Ecology – bathymetry and environments

**Figure 4:**
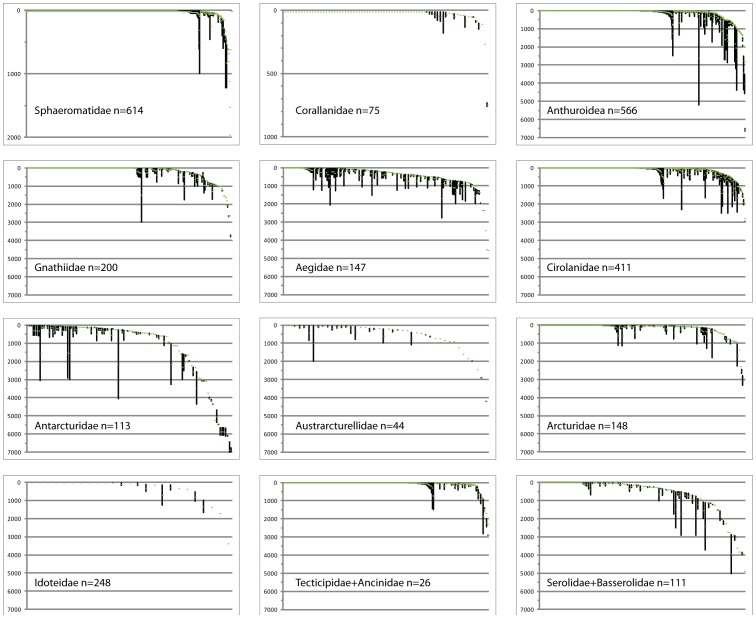
Bathymetric ranges of species of the larger families (and groups of related families) of marine isopod families. Data come from Schotte et al. [2], gaps filled by data from original publications. Species are ranked, left to right, from shallowest minimum depth to deepest, with depth records <10 m coalesced as 10 m for clarity. Numbers of species of each family or family group for which data are readily available are given. Green dots are average depths. Vertical axes are depths in metres, not to the same scale.

Isopods range from the intertidal to the depths of the oceans (Fig. 4). The maritime genera such as *Ligia* and *Tylos* belonging to Oniscidea, a generally terrestrial taxon not considered here, live above the high tide. Most species are limited to shallow-water habitats on rocky shores, muddy environments and sandy beaches. The supralittoral *Paravireia holdichi* Brökeland Wägele & Bruce, 2001 (Sphaeromatoidea) [67], *Campecopea hirsuta* (Montagu, 1804) [68] and some species *Eurydice* [69] are among the few exclusively intertidal representatives, with most intertidal species also extending to at least the shallow subtidal. The deepest recorded species is the antarcturid, *Chaetarcturus ultraabyssalis* Birstein, 1963 recorded from a trench in the NW Pacific at 6435–7280 metres but none of the families considered are as diverse or rich in species at bathyal and abyssal depths as the Asellota. Most species would appear to have a limited depth range but so few described species have been recorded more than once that this could well be questioned except on theoretical grounds or empirical evidence from other taxa [70]. Some species would appear to show considerable depth ranges (see outliers in Fig. 4) raising suspicions about the accuracy of identifications, especially for those occurring at subtidal and shelf depths as well as beyond a few hundred metres. One striking example is *Caecognathia elongata* Krøyer, 1849 from the littoral of Greenland down to 3000 m. Of the Cymothoida, more than half the known species of Cirolanidae and Gnathiidae, and three-quarters of Corallanidae and Anthuroidea have been recorded from subtidal habitats (Fig. 4). Another cymothoidan family, Aegidae, is distributed differently: many species range widely over shelf and slope depths. More than half of the species of the valviferan families Antarcturidae, Arcturidae and Austrarcturellidae are restricted to shelf depths but a significant fraction either extend to or are confined to slope and abyssal depths. The Idoteidae and Chaetiliidae differ with the vast majority confined to the immediate subtidal or inner shelf and relatively few extending to slope depths. Two-thirds of sphaeromatid species are confined to the shelf, while some extend deeper. Most species of Serolidae are shallow subtidal or shelf species and few can be called deep-water. While the patterns of family depth ranges are similar, differences are more evident at a generic level. For example, while many are strictly intertidal to subtidal (e.g., most Idoteidae) others are strictly abyssal or appearing on the shelf only in Antarctica (e.g., several Antarcturidae, *Brucerolis*).

Shallow-water isopods inhabit any suitable refugium, including sediments. Crevices, dead barnacle tests, dead mollusc shells (or fragments thereof) worm tubes, under surface of rubble or rocks, algal holdfasts, algal turf, dead wood, sand are suitable habitats. In shallow water isopods generally avoid habitats with high levels of silt, and diversity consequently drops in estuaries, mangroves and coral reef lagoon habitats. In shallow sediments (intertidal to 30 m) mobile sands and gravel are strongly preferred; diversity and densities are higher than over stable sand areas with worm tubes or sea grasses.

Sandy beaches with wave action have a characteristic and predictable suite of isopods around the world. Cirolanids are typically present, the dominant intertidal genera being *Eurydice* and *Excirolana*, Australia and New Zealand being the only exception with the representative cirolanids being species of *Pseudolana*, *Eurylana* and *Pseudaega*. In the tropics the sphaeromatid genus *Sphaeromopsis* is widespread, and species of *Exosphaeroma* occur on some beaches. Species of the valviferan genera *Chiridotea* [71], *Chaetilia* [72] and *Macrochiridothea* [73] are present on American and New Zealand beaches, and Serolidae are known from southern South American beaches.

Coral reefs, with high spatial complexity, may have the highest marine isopod diversity per unit area of any habitat, and although the reef flat and outer slope (the ‘living reef’) has a predictable suite of representative families and genera, it is too diverse to discuss in detail [74]–[77]. One characteristic that is generally true for coral reefs around the world is that Valvifera are generally rare, and when reported have usually been collected from adjacent off-reef habitats. Recent extensive and thorough collections of isopod made during the CoML–CReefs program (2008–2010) indicated that individual shelf reef regions such as Lizard Island, Great Barrier Reef, Australia, and adjacent outer reef and Heron Island, Australia, have approximately 150–200 species of isopods, not including the off-reef sea floor rubble and sediments.

Macroalgae constitute a major habitat for Limnoriidae, Sphaeromatidae, Idoteidae and Holognathidae in temperate and cool waters. In tropical and subtropical regions algal turf usually contains a restricted assemblage of species of Sphaeromatidae, Anthuroidea and some cirolanids. Certain algae such as *Sargassum* are used by some Idoteidae and certain genera of Sphaeromatidae such as *Cerceis* and *Cymodoce* though these species are not restricted to *Sargassum*. In contrast, sea grasses seem to host few isopods, the notable exception being some species of the limnoriid genus *Limnoria* particularly all three species of *Lynseia* that live as leaf miners [78], [79]. The hollow stems of seagrasses also provide the specialist caddis-like home of the holognathid genera *Cleantis* and *Cleantioides*. Another specialist plant resource is wood, notably for wood-boring ‘gribble’ of the genus *Limnoria* and for the deep-sea valviferan genus *Holognathus*.

Few species of isopod are genuinely planktonic, although rather more are bentho-planktonic, swim in the plankton when breeding or during the paratenic phase. Bentho-planktonic species occur in the cirolanid genera *Eurydice*, *Natatolana* and some Sphaeromatidae, while a paratenic phase is characteristic of the Gnathiidae [80] and Cymothoidae [81]. Genuine meso-planktonic species include *Metacirolana caeca* and *Pontogelos* (Cirolanidae), *Anuropus* (Anuropidae) and *Syscenus* (Aegidae), and typically have extensive multi-ocean distribution and lack eyes. *Barybrotes indus* (Barybrotidae) appears to be a nektonic species. Numerous species have be recorded as rafting on algae and other flotsam but the cosmopolitan *Idotea metallica* would seem to be the only obligate rafter [82].

Subtidal sediments, ranging from pebbles and gravel through to sand and mud, are rich in isopod species. Particularly rich in shallow waters are clean (largely silt free) mobile sand and gravel such as found at the base of large bommies, in the groove of ‘spur and groove’ on coral reefs, or where wave and current action keeps the sand mobile. As sediments become silt-laden families such as Cirolanidae and Sphaeromatidae decrease in diversity, while in the deep ocean (>1000 m) Asellota increase in diversity. Many anthuroids are tolerant of high mud content and survive by building tube-shelters.

Symbioses, beyond that of the parasitic Cymothoidae are not common. In the Cirolanidae the tropical monotypic genus *Cartetolana* inhabits the oral disk of certain crinoids (e.g., *Comanthus* spp.) and *Neocirolana hermitensis* inhabits shells occupied by species of the hermit crab *Dardanus*. Several species and genera of Sphaeromatidae are known to associate with sponges, the large genus *Oxinasphaera* appearing to be an obligate sponge associate [83]; *Xynosphaera colemani* burrows into the tissue of alcyonaceans; and some species of *Moruloidea* and *Cassidias* have been reported from gorgonians, their body form mimicking the shape of the polyps [84]. Antarcturids in the deep sea and arcturids at shallower depths are frequently associated with erect corals and hydroids that enable them to filter-feed up off soft sediments [85]. Black hydroids may be the obligate substrate of the tropical valviferan *Amesopous richardsonae*. Other isopods that appear to associate with sponges include some species of Aegidae and Corallanidae and also some Gnathiidae. Some Corallanidae appear to be commensals of tropical fishes, notably *Argathona macronema* (Corallanidae) and *Epulaega lethrina* (Aegidae), both species feeding on fish mucus not blood.

### Feeding

Although it is remarkable how few studies have directed research at feeding modes, four broad categories can be recognised as much on the basis of anecdotal evidence as hard data: detritus feeders and browsers, carnivores, parasites, and filter feeders. Detritus feeding is an attributed feeding category, generally applied without direct evidence to groups in which the mandible and molar process are not adapted to parasitic or carnivorous feeding. Typically the Sphaeromatidae are considered to be browsers or detritus feeders, and certainly virtually no species of that family has been shown to be a carnivorous scavenger or predator, although one species has been taken in baited trap and a small number of species appear to have incisory mandibles (e.g., *Xynosphaera*).

Carnivorous feeders can be further split into three groups—micropredators, predators and scavengers. The Cirolanidae have species that are active predators such as species of *Eurydice* and *Metacirolana*, but the majority are scavengers, including well-known examples such as giant deep-sea isopods of the genus *Bathynomus* [86]. Cirolanids are voracious scavengers, and can occur in vast numbers, and have been known to reduce a seal carcass to skin and bone overnight [87]. The Cymothooidea include seven families that show a progressive development towards parasitism, culminating in the Cymothoidae that live on the external surfaces and in the buccal and gill cavities of their fish hosts or burrow into the muscle. Carrying cymothoid parasites has been shown to result in parasitic castration [88]. The Corallanidae [89] contain genera that are commensal on fishes, live in sponges and some that are micropredators, even known (personal experience) to feed off humans; others feed on worms (e.g. *Lanocira*), and possess grasping and piercing mouthparts similar to those of the Cymothoidae. The Aegidae [47] are all micropredators of fish, taking a blood meal, and generally not staying on the host. Two aegid genera, *Rocinela* and *Syscenus*, are known to stay attached for a period, and *Syscenus* may attach permanently though this is not known for certain. Cymothoidae are all obligate parasites of fishes, and feed on host tissues and fluids at some stage of their life; they have been shown to possess anticoagulants [90], and it has been convincingly shown that these parasites depress breeding success [91] and have a castration effect on male fish [88].

Data on feeding habits of anthuroids are few but both the leptanthurid genus *Accalathura* [92] and anthurid genus *Cyathura* [93] are predators, and this is assumed for other species. Serolidae are predators [94]. Valviferans are variously algal browsers [95] or filter feeders [85], [96].

### Reproduction

Isopods retain the ova in a brood pouch, as in other peracarid crustaceans, and release offspring as mancas (juveniles resembling adults except for the absence of the last pair of pereopods), bypassing a larval phase. While gnathiids and cymothoids have a paratenic phase, praniza and aegathoid respectively, these are morphologically mancoid or immature individuals. The brood pouch is composed of oostegites arising usually from the coxae of some or all of pereopods 2–7. There are number of derived conditions including holding the ova and pre-release mancas in invaginations of the ventral body wall (e.g., *Excirolana*, some sphaeromatids) and also oostegites being lost and replaced by anterior and posterior pockets in the Sphaeromatidae [97]; some genera of Cymothoidae have a posterior fold [98].

Ova size and number of ova are directly correlated to isopod size, small species carrying few ova (<10) while large species have more and usually larger ova (the eggs of *Bathynomus* species are larger than most species of isopod). Fish-parasites have a large brood pouch, which in gill-attaching genera is sufficiently large to be described as an ‘egg sac’, carrying a very large number of eggs, and then pre-mancoid young. The number of released mancas is directly related to the size of the isopod, and ranges from 10 to 1,600 individuals [91], [99]–[101].

Males have gonopores on the medially-expanded coxal plates of pereopod 7 that cover the sternum. The pores may be separate or close together and may or may not be at the end of penial processes. In some valviverans the penes are fused into a single process. Sperm transfer is assisted by paired stylets on the inner edge of the endopod of pleopod 2. In some arcturoid valviferans the stylets form a complex interaction with highly modified and grooved first pleopods.

For most isopod species the process of sexual determination is not known. Sexes are usually separate but some are hermaphroditic. Anthuroids generally are protogynous, the terminal swimming males stage being relatively rare in a population [102]. Cymothoidae are obligate parasites of fishes. In those genera that attach in the mouth, gills of body cavity, a small ‘dwarf’ male is usually associated with a large female. In the externally attaching genera such as *Anilocra* and *Nerocila* individuals are protandrous and less likely to occur in pairs; *Renocila* is an exception.

Strong sexual dimorphism is characteristic of several isopod families, evident most simply in differences in body proportions; females are wider than males in idoteids but narrower in serolids. The pereopods of males of these two families may be more setose than females and males of serolids may have pereopods modified for coitus [103]. The most extreme differences are seen in Gnathiidae, the adult males of which have obvious and often large mandibles projecting anteriorly on a somewhat quadrate and robust head. Females in contrast have a small and anteriorly rounded or narrowed head and inflated pereon [56], [104]. Female Cymothoidae are two to three times as large as their accompanying males, and usually have smaller eyes; the males have a simple bilateral body shape, while females may be axially twisted and have mores strongly developed coxal plates, and may show carinae and lobes on the pereopods that are absent in the male. Sphaeromatidae are often strongly dimorphic, with males showing a high degree of cuticular ornamentation, including prominent spines, variously perforate pleotelson shapes and variously reduced or expanded uropods, whereas in contrast the females present what can be called a simple morphology [105]. For most families the difference is in the primary sexual characters and often the antennule and antenna (more heavily setose in males). In a small number of genera such as *Metacirolana* and *Eurydice* and all anthuroids, males undergo a change into a ‘swimming male’ morphology with enlarged eyes, reduced mouthparts, a more elongate pleon, and the antennule more elongate and with numerous aesthetascs.

Neoteny is a feature of some anthuroid genera, notably within Paranthuridae [106], and is seen also in some deep-sea asellotes.

A haremic breeding structure is known in some Sphaeromatidae [107]–[109] and all Gnathiidae [80], [110].

### Biogeography

About half of all coastal, shelf and upper bathyal species have been recorded in temperate realms, 40% in tropical regions and the remainder in polar seas (Table 2, Fig. 2A). The greatest concentration of temperate species is in Australasia; more have been recorded from temperate North Pacific than the assumedly better studied North Atlantic. Of tropical regions, the Central Indo-Pacific is home to more species any other region. This is consistent with findings for many other taxa where this region is referred to as the Coral Triangle [111], a centre with extreme species richness from whence diversity declines in all directions but especially into the central Pacific. The numbers of species in non-tropical regions are decidedly asymmetrical latitudinally with 1.35 times as many species in temperate Southern Hemisphere than the temperate North Atlantic and northern Pacific, and almost four times as many Antarctic as Arctic species, as has been long demonstrated [112], [113]. Difference in sampling effort can not be invoked to explain such differences and our experience in Australasia demonstrates that the asymmetry is greater than the data suggest (see next section). Family dominance was not the same from realm to realm. Valviferans dominated in polar regions, Arcturidae and Idoteidae in the Arctic and Antarcturidae in the Antarctic while these taxa were virtually absent from all tropical regions (except the Tropical Atlantic). The families that dominated in tropical regions (relative to other regimes) are Cirolanidae, Cymothoidae, Anthuridae, Expanathuridae and Leptanthuridae, all predatory or associated in some way with fishes. On the other hand, temperate regions are more favourable for Idoteidae and Sphaeromatidae. Only one large family is endemic to a realm, Holidoteidae in Temperate Southern Africa. Three valviferan families Pseudidotheidae, Rectarcturidae and Xenarcturidae with only five species in total, are found only in Gondwanan continents. Austrarcturellidae concentrated in one realm, Temperate Southern Australasia (all exceptions are southern hemisphere). Basserolidae and Serolidae are concentrated in Antarctica, Temperate Southern Australasia and South America, and Central Indo-Pacific with outliers in deeper water elsewhere.

Sampling in deeper waters has been more haphazard and data rely on few expeditions (Table 3, Fig. 2B); The taxa under consideration are less abundant and diverse in these provinces than are the Asellota [114]–[116]. Generalisations are that more species are known from the deep Antarctic than Arctic, and more from the larger Pacific than Atlantic oceans. Two areas with many species known are the New Zealand-Kermadec province, thanks to the work of several Australian workers, and the Northern North Pacific, thanks to the work of Kussakin [117], [118]. Antarcturidae and Serolidae are the most species-rich families, especially in the Southern Hemisphere while Gnathiidae are common in all oceans. The absence of species in the southeastern Pacific Region and presence of only two in the North Pacific surely indicates absence of sampling, especially compared to other Pacific regions.

### Sampling and taxonomic gaps

Shallow depths (intertidal to shelf) of some regions have received considerable attention and may be considered well understood with few species remaining to be discovered: Europe [119] and Scandinavia [120], eastern and western North America [19], perhaps also Antarctica [121], Caribbean Sea [122], the Australian Great Barrier Reef (papers by Bruce and Poore), and South Africa [123]. Many areas have received no sustained taxonomic attention and remain very poorly known, including most of the Indian Ocean in spite of the efforts of B. Kensley and coauthors [124]–[128], the west coast of Africa, South America, the Pacific islands [129]–[131], and the Indo-Malaysian triangle including Indonesia and the Philippines [132], the latter a region of known high marine diversity.

Others areas have been well sampled by locally based taxonomists but remain only partially described. These have yielded extensive collections now in museums awaiting description. Notable among these is south-eastern Australia where the continental shelf and slope serolid, sphaeromatid and antarcturid fauna is known to contain dozens of undescribed species. Western and northern Australia are generally poorly explored.

While shallow and easily accessed habitats close to civilisation have been the centre of taxonomic study, soft sediments in the deep sea have also received considerable attention, especially recently in the Atlantic Ocean and Weddell Sea [116], [133]. The richness of asellotes has been the principal finding. Broad ranging studies on deep-sea non-asellotes are fewer, exceptions being in Antarctica by Brandt on Serolidae, Cirolanidae and Valvifera [134], [135] and Schultz on Valvifera [136], in the North Pacific by Kussakin [117] and the Atlantic by Menzies [24]. Other deep-sea habitats are more difficult to sample, notably hard substrates such as found on seamounts and the steep slopes surrounding the Pacific high islands. Such samples from these habitats that do exist suggest an as yet undocumented fauna.

Cryptic species of Crustacea, those that have so-far not been distinguished morphologically, have been revealed in increasing numbers recently following molecular investigation. Few examples exist for Isopoda, Held's [54], [137] studies of *Glyptonotus* and *Ceratoserolis* being exceptions and where morphological differences can be found post hoc. Even without the assistance of molecular evidence, species swarms are known to us, e.g., in the sphaeromatid *Oxinasphaera* [48] and cassidiniines [138]. The *Cirolana parva*-group is known to be similar, currently about 26 or 27 closely similar species, with at least as many species again in collections, and probably double that still to be collected. Several smaller but similar groups exist in all large cirolanid genera, *Bathynomus*, *Eurydice*, *Metacirolana*, *Natatolana*, and *Cirolana*. In Sphaeromatidae cryptic species swarms are suspected within most large genera: *Cilicaeopsis*, *Cilicaea*, *Paracilicaea*, *Cymodoce*, *Dynamene*, *Dynamenella*, *Dynoides*, *Exosphaeroma*, *Gnorimosphaeroma*, *Pseudosphaeroma* and *Paracassidina*. The aegid genera *Aega* and *Aegiochus* and anthuroid genera *Leptanthura* and *Paranthura* are equally diverse.

Confidence in estimates of the numbers of undescribed species is low. We estimate, largely on the basis of fractions of known species in samples from new environments, that around one fifth of all species in intertidal-upper slope habitats are known. Poore et al. [139] reported 78% of 110 species in the non-asellote taxa sampled on the slope as new, a figure that remains little changed today. Recent sampling in similar environments in Western Australia has found 83% of 47 non-asellote species as new [Poore, unpublished]. Incidentally, percentages for asellotes are much higher. Applying this figure to the number so far described from these environments a global estimate of ∼14300 species of non-asellotes is reached. This could be perhaps multiplied by 2–5 to account for cryptic species reaching 28500–71000. Exploration of the deep sea is less advanced and the 200 species so far described could be only a sample of perhaps ten times as many, i.e. another 2000. In total an estimate for these taxa of 30500–73000 species. Using different methods and starting point our estimate for WoRMS (Appeltans et al. submitted) for all marine isopods was 83000, a figure that may have to be revised upwards if the fraction of asellotes remains around half of all marine isopods.

### Human issues – economic and environmental impact

Bird [140] described how the Florida shark fishery collapsed when cirolanids (*Natatolana* spp.) swarmed over one summer, eating their way into the living sharks and destroying their vital organs so causing death. In New Zealand and Australia [141], [142] cirolanids have been identified as attacking fish caught both in fish traps and trawl nets, at times rendering the fish unsaleable. In ‘olden days’ charts were marked as ‘lousy ground’ as indication that there was the potential for swarming cirolanids and therefore a place to be avoided by fishers. Cirolanids have been further used by the shark cartilage industry cleaning the shark carcasses of flesh prior to processing.

Isopods have only occasionally featured as a diet item for humans, with anecdotal accounts of *Bathynomus* being eaten in the Caribbean and *Ligia* being occasionally eaten by Polynesians. Medicinal properties have occasionally been attributed to isopods, in the marine context the only reference to our knowledge is that of curative properties attributed to ‘fiske bjørn’ (*Aega* spp.) by the ancient Nordics, specifically the dried blood-filled gut.

Species of *Limnoria*, ‘gribble’ were notorious for boring into and damaging wharf and ship timber along with two or three species of *Sphaeroma* [143]. Their effect on marine and estuarine timbers became less serious with the advent of treated timbers, although they are still a problem if not monitored (e.g., New Zealand railway bridge collapse [144]. These species too are examples of translocation in the hulls of wooden ships and some species are now widespread [145]. Other isopods have also been transported more recently, e.g., *Cirolana harfordi*, *Paradella dianae* and *Paracerceis sculpta* to Australia [146] and *Pseudosphaeroma* within Australasia [147]. Another is *Synidotea laticauda* from San Francisco, USA, to Europe [148] but misidentifications of species of *Synidotea* have lead to erroneous reports of widespread translocation of the Japanese species *S. laevicaudata* [149].

The marine isopod *Bathynomus giganteus* remains one of the largest mobile marine crustaceans, subject of some wonderment in the popular press and on the web. Accounts of individuals 30 inches long may be far fetched but even at 365 mm in length the species is a voracious and impressive scavenger in the tropical western Atlantic.
