# Correction: Global Diversity of Marine Isopods (Except Asellota and Crustacean Symbionts)

**DOI:** 10.1371/annotation/3260cd00-89cf-4e08-ab25-07e0be598ab4

**Published:** 2012-09-19

**Authors:** Gary C. B. Poore, Niel L. Bruce

There were errors in the placement of Figures 1, 2, and 3. The figure currently labeled as Figure 1 is actually Figure 2. The figure currently labeled as Figure 2 is actually Figure 3. The figure currently labeled as Figure 3 is actually Figure 1. The legends are in the correct locations.

The dates of receipt and acceptance of the manuscript are missing. They are: Received: March 1 2012, Accepted: July 23 2012. 

